# Hypokalemia Events With Sodium Zirconium Cyclosilicate and Placebo in Hemodialysis Patients

**DOI:** 10.1016/j.ekir.2022.01.1058

**Published:** 2022-01-28

**Authors:** Steven Fishbane, Martin Ford, Masafumi Fukagawa, Kieran McCafferty, Anjay Rastogi, Bruce Spinowitz, Konstantin Staroselskiy, Konstantin Vishnevskiy, Vera Lisovskaja, Ayman Al-Shurbaji, Nicolas Guzman, Sunil Bhandari

**Affiliations:** 1 Department of Medicine, Zucker School of Medicine at Hofstra/Northwell, Great Neck, New York, USA; 2 Department of Renal Medicine, King’s College Hospital NHS Trust, London, UK; 3 Faculty of Life Sciences and Medicine, King’s College London, London, UK; 4 Division of Nephrology, Endocrinology and Metabolism, Department of Internal Medicine, Tokai University School of Medicine, Isehara, Japan; 5 Department of Nephrology, Barts Health NHS Trust, London, UK; 6 UCLA CORE Kidney Program, University of California, Los Angeles, Los Angeles, California, USA; 7 Department of Medicine, New York-Presbyterian Queens, Queens, New York, USA; 8 Department #2, B. Braun Avitum Russland Clinics, St. Petersburg, Russia; 9 Department of Internal Medicine, Clinical Pharmacology and Nephrology, North-Western State Medical University named after I.I. Mechnikov, St. Petersburg, Russia; 10 Biometrics and Information, AstraZeneca BioPharmaceuticals Research and Development Gothenburg, Mölndal, Sweden; 11 Global Medicines Development, AstraZeneca BioPharmaceuticals Research and Development Gothenburg, Mölndal, Sweden; 12 Global Medicines Development, AstraZeneca BioPharmaceuticals Research and Development, Gaithersburg, Maryland, USA; 13 Department of Renal and Transplant Medicine, Hull University Teaching Hospitals NHS Trust, Hull, UK

Lowering predialysis serum potassium (K^+^) concentration is an important function of hemodialysis in patients with end-stage kidney disease, as a component for the effective management and avoidance of hyperkalemia. Because of the typical K^+^ removal that occurs, serum and total body K^+^ are at their nadir immediately after hemodialysis; postdialysis hypokalemia is reported to occur in 35% to 45% of patients, which may incur additional risks.^1^, ^2^, ^3^

The clinical importance of hypokalemia in end-stage kidney disease is under-recognized.^3^^,^^4^ Patients with combined predialysis and postdialysis hypokalemia are at significantly higher mortality risk versus patients without either predialysis or postdialysis hypokalemia.^4^ Predialysis hypokalemia occurring independently is associated with increased mortality risk^4^; for postdialysis hypokalemia, mortality risk seems to be dependent on predialysis serum K^+^ concentration.^4^

Sodium zirconium cyclosilicate (SZC) is a novel, highly-selective K^+^ binder approved for the treatment of hyperkalemia in adults, including those undergoing maintenance hemodialysis.^5^, ^6^, ^7^, ^8^ The phase 3b DIALIZE study (NCT03303521) revealed that SZC is an effective and well-tolerated treatment for predialysis hyperkalemia in patients with end-stage kidney disease.^9^ In DIALIZE, events of predialysis hypokalemia (serum K^+^ concentration < 3.5 mmol/l) were reported in 5.1% of placebo patients and 5.2% of SZC patients (both *n* = 5).^9^ In this *post hoc* safety analysis, we report a detailed analysis of hypokalemia events from DIALIZE (Supplementary Methods).

## Results

### Patients

The DIALIZE safety analysis set comprised 195 patients (SZC *n* = 96, placebo *n* = 99). At baseline, overall mean (SD) age was 58.2 (13.7) years, 58.5% of patients were male, and mean (SD) weight was 74.0 (19.5) kg (Supplementary Results and Supplementary Table S1).

### Predialysis Hypokalemia

There were 12 (SZC *n =* 7, placebo *n* = 5) registered instances of predialysis hypokalemia (Table 1). Predialysis hypokalemia occurred at a higher numerical frequency during the 4-week evaluation period for patients receiving SZC (5 instances, 4.2% of patients) versus placebo (2 instances, 2.0% of patients) (Supplementary Figure S1A and Table 1). Instances occurred at long interdialytic interval and short interdialytic interval visits in both treatment arms (Table 1). Predialysis serum K^+^ concentrations by study visit among patients with predialysis hypokalemia are presented in Supplementary Figure S1B.

**Table 1:** Registered instances of predialysis and postdialysis hypokalemia

Study visit type	Study period	SZC (*N* = 96)		Placebo (*N* = 99)	
		Number (%) of patients^a^	Number of instances	Number (%) of patients^a^	Number of instances
		Predialysis hypokalemia			
LIDI or SIDI	Overall	5 (5.2)	7	5 (5.1)	5
	Screening	0	0	0	0
	Dose titration	2 (2.1)	2	2 (2.0)	2
	Evaluation	4 (4.2)	5	2 (2.0)	2
	Follow-up	0	0	1 (1.0)	1
LIDI	Overall	3 (3.1)	3	2 (2.0)	2
	Screening	0	0	0	0
	Dose titration	1 (1.0)	1	0	0
	Evaluation	2 (2.1)	2	2 (2.0)	2
SIDI	Overall	2 (2.1)	4	2 (2.0)	2
	Screening	0	0	0	0
	Dose titration	1 (1.0)	1	2 (2.0)	2
	Evaluation	2 (2.1)	3	0	0
		Postdialysis hypokalemia			
LIDI or SIDI or follow-up	Overall	75 (78.1)	397	57 (57.6)	239
	Dose titration	64 (66.7)	205	49 (49.5)	131
	Evaluation	61 (63.5)	166	43 (43.4)	88
	Follow-up	26 (27.1)	26	20 (20.2)	20
LIDI	Overall	74 (77.1)	339	55 (55.6)	195
	Dose titration	63 (65.6)	173	46 (46.5)	107
	Evaluation	61 (63.5)	166	43 (43.4)	88
SIDI	Overall	16 (16.7)	32	15 (15.2)	24
	Dose titration	16 (16.7)	32	15 (15.2)	24
	Evaluation	0 (0.0)	0	0 (0.0)	0

K^+^, potassium; LIDI, long interdialytic interval; SIDI, short interdialytic interval; SZC, sodium zirconium cyclosilicate.

Safety analysis set (*N* = 195). Percentages are based on the total numbers of patients in the treatment group (*N*). Only LIDI (study visits 4, 7, 9, 10, 11, 12, 13, 14, and 15), SIDI (study visits 5, 6, 7.5, 8, 9.5, 10.5, 11.5, 12.5, 13.5, and 14.5), and follow-up (study visit 16) included. No postdialysis serum K^+^ measurements were collected during screening. Study period was derived from visit. Dose titration was defined as after screening and before visit 12; evaluation was defined as on and after visit 12 and before visit 16; follow-up period was defined as visit 16.

a Number (%) of patients with predialysis or postdialysis hypokalemia (serum K^+^ concentration of <3.5 mmol/l).

### Severe Predialysis Hypokalemia

A total of 3 patients (SZC *n* = 2, placebo *n* = 1) each reported 1 event of severe predialysis hypokalemia (serum K^+^ concentration < 2.7 mmol/l), occurring while in a dialysis clinic. Event summaries for patients 1 (placebo), 2 (SZC), and 3 (SZC) are described in the Supplementary Results, and predialysis serum K^+^ profiles are plotted in Supplementary Figure S2. As predialysis serum K^+^ samples were evaluated using a central laboratory, study investigators were not aware of a patient’s status of severe hypokalemia at the time of measurement. As such, patients with instances of severe predialysis hypokalemia were initiated on dialysis as per their routine care. The patients completed their dialysis treatment and returned home. No associated adverse event was reported with any instance of severe predialysis hypokalemia.

### Postdialysis Hypokalemia

Overall, 78% of SZC (*n* = 75) and 58% of placebo (*n* = 57) patients reported instances of postdialysis hypokalemia (Table 1). Proportions of patients with postdialysis hypokalemia at each study visit were numerically greater with SZC than placebo, between 24% to 46% and 15% to 27%, respectively, during the titration period and 43% to 51% and 19% to 29%, respectively, during the evaluation period (Supplementary Figure S3A). Events occurred at long interdialytic interval and short interdialytic interval visits with both SZC and placebo (Table 1). Among patients with postdialysis hypokalemia, median and range (minimum–maximum) values of postdialysis serum K^+^ concentration across study visits were generally comparable between treatment groups, between 3.20 to 3.35 mmol/l (2.00–3.4 mmol/l) with SZC and 3.25 to 3.30 mmol/l (2.00–3.4 mmol/l) with placebo (Supplementary Figure S3B).

In both arms, greater proportions of patients with lower baseline dialysate K^+^ concentrations had postdialysis hypokalemia (dialysate K^+^ 1 mmol/l: SZC 100% [*n* = 2], placebo 100% [*n* = 1]; 2 mmol/l: SZC 90% [*n* = 60], placebo 73% [*n* = 51]) versus those with baseline dialysate K^+^ concentration of 3 mmol/l (SZC 48% [*n* = 13], placebo 15% [*n* = 4]) (Supplementary Table S2).

**Figure 1:**
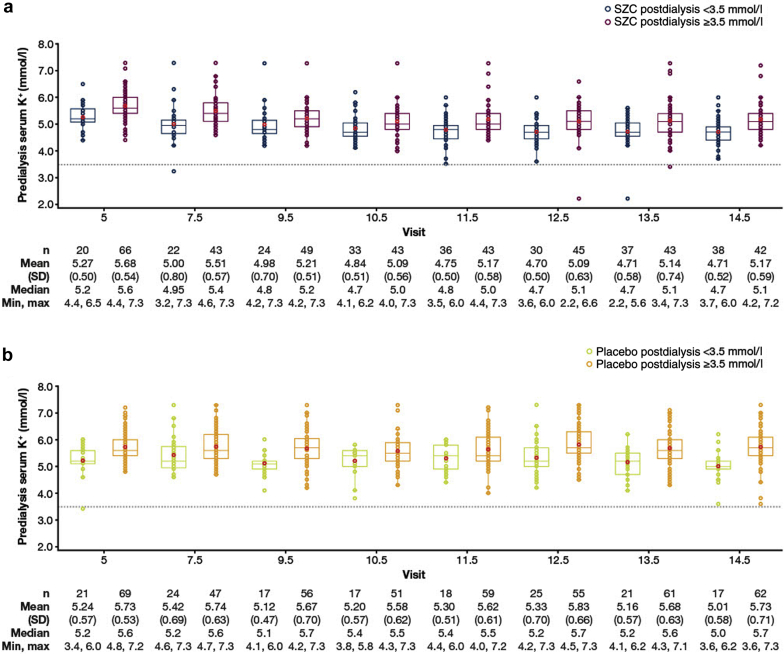
Box and whisker plot of predialysis serum K^+^ concentrations at subsequent SIDI visits, stratified by postdialysis serum K^+^ status at the previous LIDI visit: (a) SZC and (b) placebo treatment arms. Safety analysis set (*N* = 195). Individual patient cases are presented. Box lines represent the Qs (Q1, Q2 [median], Q3), red symbols within the boxes represent mean values, whiskers extend to the last values within ±1.5 × (Q3–Q1), and symbols beyond the whiskers represent extreme values. Hypokalemia was defined as serum K^+^ concentration of <3.5 mmol/l (gray dashed line). Values found are predialysis serum K^+^ concentrations at SIDI study visits that followed a LIDI, stratified by postdialysis serum K^+^ status (≥3.5 vs. <3.5 mmol/l) at the previous visit. Visits 5, 7.5, 9.5, 10.5, 11.5, 12.5, 13.5, and 14.5 are SIDI visits, consistent with the study design of DIALIZE.^9^ K^+^, potassium; LIDI, long interdialytic interval; Max, maximum; Min, minimum; Q, quartile; SIDI, short interdialytic interval; SZC, sodium zirconium cyclosilicate.

For all but 2 SZC patients with postdialysis hypokalemia at a long interdialytic interval visit, predialysis serum K^+^ concentration returned to ≥3.5 mmol/l at the next study visit (Figure 1a). In both arms, predialysis serum K^+^ concentrations were generally higher for patients with postdialysis serum K^+^ concentration ≥ 3.5 mmol/l versus <3.5 mmol/l at the previous study visit (Figure 1a and b).

### Patients With Combined Predialysis and Postdialysis Hypokalemia by Study Visit

Events of combined predialysis and postdialysis hypokalemia at the same study visit were low in both treatment arms (both *n* = 1 [1.0%]). Both events occurred during the 4-week evaluation period (Supplementary Results).

## Discussion

In our *post hoc* analysis, the overall number of events of predialysis hypokalemia was low, and the proportions of patients overall with predialysis hypokalemia were similar with SZC and placebo. Few patients (SZC *n* = 2, placebo *n* = 1) in the study had instances of severe predialysis hypokalemia, and instances were not associated with any adverse events.

We observed no increased risk of combined predialysis and postdialysis hypokalemia with SZC versus placebo. This finding is important as the combination of predialysis and postdialysis hypokalemia has been found to be associated with the highest mortality risk versus patients without either predialysis or postdialysis hypokalemia.^4^

A greater proportion of SZC patients had postdialysis hypokalemia versus placebo, consistent with the mechanism of action of SZC; however, the severity, as measured by median and range of postdialysis serum K^+^ concentration, was comparable between treatment arms. In patients undergoing maintenance hemodialysis, postdialysis hypokalemia is usually transient—in the hours after dialysis, serum K^+^ concentration rapidly rebounds because of continued redistribution of K^+^ from the intracellular to the extracellular space. Indeed, after instances of postdialysis hypokalemia in DIALIZE, serum K^+^ concentration rebounded to within the clinically acceptable range by the next short interdialytic interval visit in all but 2 SZC patients. In DIALIZE, postdialysis serum K^+^ measurements were mostly collected at long interdialytic interval visits during the treatment period. Therefore, postdialysis serum K^+^ data are enriched for time points when serum K^+^ concentrations are highest, which most likely explains the lower rates of postdialysis hypokalemia (19%–29%) observed in the placebo arm at each study visit than in the literature (35%–45%).^1^^,^^2^ Finally, higher rates of postdialysis hypokalemia among patients with lower baseline dialysate K^+^ concentrations (1–2 mmol/l) in both arms likely reflect greater reductions in serum K^+^ achieved because of the higher serum K^+^ gradient, although low patient numbers in the dialysate K^+^ at 1 mmol/l group precluded reliable estimation.

The present analyses have several limitations. The analyses are *post hoc* in nature and were not prespecified. Therefore, the results are exploratory and hypothesis generating. Second, postdialysis serum K^+^ concentration was taken immediately after dialysis sessions and measured using central laboratory assessment; therefore, persisting and incident postdialysis hypokalemia could not be fully distinguished. Finally, the association of hypokalemia events and adverse clinical outcomes was not explored.

In conclusion, these *post hoc* analyses provide additional evidence on the safety of SZC in the management of hyperkalemia in patients with end-stage kidney disease. Despite the efficacy of SZC in lowering predialysis serum K^+^ concentration, these descriptive analyses suggest that SZC was not associated with a clinically significant increase in the frequency of predialysis hypokalemia. Treatment with SZC, versus placebo, did not increase the frequency of combined predialysis and postdialysis hypokalemia at the same visit, which is associated with increased mortality risk. Although postdialysis hypokalemia was more frequent with SZC than placebo, it did not persist to the next study visit for all but 2 patients in the SZC arm.

## Disclosure

SF reports receiving research support and consulting fees from AstraZeneca. MFo reports receiving travel support from Amgen and AstraZeneca and is an advisory board member for AstraZeneca. MFu reports receiving consulting and lecture fees from AstraZeneca Japan. KM reports being an academic grant holder and advisory board member for AstraZeneca. AR reports receiving research or travel support from and/or is a speaker, consultant, or advisory board member for AstraZeneca, Relypsa, Fresenius Medical Care, Sanofi, Kadmon, AMAG, Otsuka, Genzyme, GSK, Omerus, Janssen, Reata Pharmaceuticals, Ironwood, and Amgen. BS reports receiving research grants, lecture fees, and/or consulting fees from AstraZeneca, Akebia, Reata Pharmaceuticals, and Fresenius Medical Care. KS reports receiving research support from AstraZeneca. KV reports receiving research support from AstraZeneca. VL, AAS, and NG are employees of AstraZeneca. SB has given lectures and participated in an advisory board for AstraZeneca, has given lectures sponsored by Vifor Pharma, and has received travel support from AstraZeneca and Vifor Pharma.

## Supplementary Material

**Supplementary Methods.**

**Supplementary Results**.

**Table S1.** Patients’ demographics and baseline characteristics.

**Table S2.** Proportion of patients with postdialysis hypokalemia events postrandomization by baseline dialysate K^+^ concentration in the SZC and placebo treatment groups.

**Figure S1.** Predialysis hypokalemia by study visit: (a) proportions of patients and (b) predialysis serum K^+^ concentrations among patients with predialysis hypokalemia.

**Figure S2.** Predialysis serum K^+^ concentration at each study visit for patients with severe predialysis hypokalemia (*n* = 3).

**Figure S3.** Postdialysis hypokalemia by study visit: (a) proportions of patients and (b) median and range of postdialysis serum K^+^ concentration.

**Supplementary References.**
