# Supplementary material for: Hypokalemia Events With Sodium Zirconium Cyclosilicate and Placebo in Hemodialysis Patients
Source: Kidney Int Rep. 2022 Jan 28;7(4):908–12. doi: 10.1016/j.ekir.2022.01.1058 (PMC9039482; doi:10.1016/j.ekir.2022.01.1058)
Supplement: Supplementary File (PDF) [file mmc1.pdf]

Supplementary Materials

Hypokalemia Events with Sodium Zirconium Cyclosilicate and Placebo in Hemodialysis Patients

Steven Fishbane[1], Martin Ford[2,3], Masafumi Fukagawa[4], Kieran McCafferty[5], Anjay Rastogi[6], Bruce Spinowitz[7], Konstantin Staroselskiy[8], Konstantin Vishnevskiy[9], Vera Lisovskaja[10], Ayman Al-Shurbaji[11], Nicolas Guzman[12], Sunil Bhandari[13]

[1]Department of Medicine, Zucker School of Medicine at Hofstra/Northwell, Great Neck, NY, USA; [2]Department of Renal Medicine, King's College Hospital NHS Trust, London, UK; [3]Honorary Senior Lecturer, Faculty of Life Sciences and Medicine, King's College, London, UK; [4]Division of Nephrology, Endocrinology and Metabolism, Department of Internal Medicine, Tokai University School of Medicine, Isehara, Japan; [5]Department of Nephrology, Barts Health NHS Trust, London, UK; [6]UCLA CORE Kidney Program, University of California, Los Angeles, Los Angeles, CA, USA; [7]Department of Medicine, NewYork-Presbyterian Queens, Queens, NY, USA; [8]Department #2, B. Braun Avitum Russland Clinics, St. Petersburg, Russia; [9]Department of Internal Medicine, Clinical Pharmacology and Nephrology, North-Western State medical university named after I.I. Mechnikov, St. Petersburg, Russia; [10]Biometrics and Information, AstraZeneca BioPharmaceuticals Research and Development Gothenburg, Mölndal, Sweden; [11]Global Medicines Development, AstraZeneca BioPharmaceuticals Research and Development Gothenburg, Mölndal, Sweden; [12]Global Medicines Development, AstraZeneca BioPharmaceuticals Research and Development, Gaithersburg, MD, USA; [13]Department of Renal and Transplant Medicine, Hull University Teaching Hospitals NHS Trust, Hull, UK

Supplementary Methods

Study Design and Patients

Full details of the DIALIZE study have been previously reported[1] and are described briefly here. DIALIZE was a randomized, double-blind, placebo-controlled, Phase 3b study conducted across Japan, Russia, the United States, and the United Kingdom. The study consisted of an 8-week treatment period, comprising 4 weeks for sodium zirconium cyclosilicate (SZC) and placebo dose titration (Days 1–29, Visits 4–11), and a 4-week evaluation period on stable dose (Days 30–57, Visits 11.5–15). Patients were randomized 1:1 to receive orally a starting dose of 5 g of SZC or placebo once daily on non-dialysis days (4 days/week). During dose titration, doses of SZC and placebo were adjusted weekly over 4 weeks to attain a pre-dialysis serum potassium (K^+) concentration of 4.0–5.0 mmol/L after the long interdialytic interval (LIDI). Doses of SZC and placebo were titrated in 5 g increments to a maximum dose of 15 g once daily on non-dialysis days. Doses could be reduced by 5 g if serum K^+ concentration was <4.0 mmol/L as a precaution against pre-dialysis hypokalemia (serum K^+ concentration of <3.5 mmol/L).

Eligible patients were adults aged ≥18 years with end-stage kidney disease who were managed for ≥3 months by hemodialysis three times weekly before randomization. During screening, patients were required to have persistent hyperkalemia despite maintenance hemodialysis, defined as pre-dialysis serum K^+ concentration of >5.4 mmol/L after the LIDI on Day –7, and pre-dialysis serum K^+ concentration of >5.0 mmol/L after at least one short interdialytic interval (SIDI) on Days –5 and –3.

Assessments

Samples of K^+ were measured using central laboratory assessment and a point-of-care i-STAT device (Abbott Point of Care, Inc., Princeton, NJ, USA), which has been shown to produce accurate, reliable, and robust K^+ measurements.[2,3] Dose adjustments were based on pre-dialysis K^+ concentration measured by i-STAT to avoid delays in treatment decision-making. For the primary analyses in DIALIZE, central laboratory serum K^+ samples were obtained pre- and post-dialysis at LIDI visits, and pre-dialysis only at SIDI visits throughout the study. Following a protocol amendment, at the discretion of study investigators, post-dialysis serum K^+ measurements were also assessed for monitoring purposes at SIDI visits in the first 2 weeks of the dose-titration period, when more pronounced changes in pre- and post-dialysis serum K^+ concentration were expected to occur. Post-dialysis serum K^+ concentrations were taken immediately following dialysis and were measured using central laboratory assessment, as per the study protocol.

***Post-hoc* Analyses**

Post-hoc analyses of hypokalemia events in the SZC and placebo arms comprised: derivations of the numbers and proportions and serum K^+ concentrations of patients with pre- or post-dialysis hypokalemia (serum K^+ concentration of <3.5 mmol/L) or combined pre- and post-dialysis hypokalemia at the same study visit, at any point during the study and by study visit; case profiles and pre-dialysis serum K^+ concentrations over time for patients who had severe pre-dialysis hypokalemia at any point during the study (defined as serum K^+ concentration of <2.7 mmol/L, as per the study protocol); pre-dialysis serum K^+ concentrations at the subsequent SIDI visit, stratified by post-dialysis serum K^+ status (≥3.5 versus <3.5 mmol/L) at the

previous LIDI visit; and proportions of patients with post-dialysis hypokalemia stratified by baseline dialysate K^+ concentration (1, 2, or 3 mmol/L) and treatment arm, with a pairwise comparison of post-dialysis hypokalemia between baseline dialysate K^+ concentration categories in the two treatment arms separately.

Post-hoc analyses were conducted on the safety analysis set (i.e., all randomized patients who received one or more doses of trial medication; n = 195). No imputation of missing data was conducted. The proportions of patients with hypokalemia (pre-dialysis, post-dialysis overall and by baseline dialysate K^+ concentration, or both pre- and post-dialysis at the same study visit) were summarized descriptively using percentages. Overall proportions (at any timepoint during the study) of patients with hypokalemia were calculated using the number of patients in each treatment arm as the denominator; proportions of patients at each study visit were calculated using the number of patients with non-missing serum K^+ measurements at the particular visit as the denominator. Serum K^+ concentrations and profiles were plotted descriptively as figures (individual values or median plus minimum–maximum range of values). Pre-dialysis serum K^+ concentrations at subsequent SIDI visits, stratified by post-dialysis serum K^+ status at the previous LIDI visit, were shown using box and whisker plots summarizing the following statistics: quartiles (Q1, Q2 [median], Q3), means, last values within ±1.5 × (Q3–Q1), and extreme values. For the pairwise comparison of post-dialysis hypokalemia between any two baseline dialysate K^+ concentration categories within each treatment arm, 95% confidence intervals (CI) for the difference were calculated using the exact method based on a score statistic.

Supplementary Results

Patients

Patient characteristics were generally similar between treatment arms, except for a small difference in age distribution: patients in the SZC group were younger (mean age [SD] = 55.9 [13.8] years) than those in the placebo group (60.4 [13.2] years) (**Supplementary Table S1**).

Severe Pre-dialysis Hypokalemia

In the SZC arm, Patients 2 and 3 had instances of severe pre-dialysis hypokalemia at a SIDI visit (both had serum K^+ concentration of 2.2 mmol/L). For both patients, pre-dialysis serum K^+ values obtained at previous and subsequent study visits were within the normal range (**Supplementary Figure S2**). Patient 2 (Visit 1 pre-dialysis serum K^+ concentration = 5.9 mmol/L) had an event of severe pre-dialysis hypokalemia in the 4-week evaluation period on Visit 12.5 (Day 38; **Supplementary Figure S2**). The patient's dose was increased to 10 g SZC after the first week in the study and was unchanged thereafter, throughout the event of severe pre-dialysis hypokalemia and until the patient completed the study. Patient 3 (Visit 1 pre-dialysis serum K^+ concentration = 5.7 mmol/L) had an event of severe pre-dialysis hypokalemia during the 4-week evaluation period on Visit 13.5 (Day 45; **Supplementary Figure S2**). The patient's dose of SZC was temporarily increased to 10 g during the titration period on Visit 7 (Day 8), before being maintained at 5 g for the 4-week evaluation period. An interruption of one dose of SZC occurred during the 4-week evaluation period on Visit 12 (Day 36), unrelated to the instance of severe pre-dialysis hypokalemia. Treatment with SZC was discontinued after the

patient was hospitalized with an adverse event deemed unrelated to the study drug by investigators.

In the placebo arm, the event of severe pre-dialysis hypokalemia (serum K^+ concentration of 2.3 mmol/L) in Patient 1 (Visit 1 pre-dialysis serum K^+ concentration = 6.9 mmol/L) occurred in the follow-up period (Visit 16, Day 71; Supplementary **Figure S2**). Patient 1 (placebo) had a post-dialysis serum K^+ concentration of 3.9 mmol/L at the same visit as their event of severe pre-dialysis hypokalemia, suggesting that their central laboratory pre- and post-dialysis serum K^+ samples could have been mis-labelled.

Patients with Combined Pre- and Post-dialysis Hypokalemia by Study Visit

For the SZC patient, combined pre- and post-dialysis hypokalemia at the same study visit occurred on Visit 14 (Day 50); pre- and post-dialysis serum K^+ concentrations were 2.7 mmol/L and 3.1 mmol/L, respectively. For the placebo patient, combined pre- and post-dialysis hypokalemia at the same study visit occurred on Visit 15 (Day 57); pre- and post-dialysis serum K^+ concentrations were 3.3 mmol/L and 3.1 mmol/L, respectively.

Supplementary References

1. Fishbane S, Ford M, Fukagawa M, et al. A phase 3b, randomized, double-blind, placebo-controlled study of sodium zirconium cyclosilicate for reducing the incidence of predialysis hyperkalemia. *J Am Soc Nephrol.* 2019;30:1723-1733.

2. Papadea C, Foster J, Grant S, et al. Evaluation of the i-STAT Portable Clinical Analyzer for point-of-care blood testing in the intensive care units of a university children's hospital. *Ann Clin Lab Sci.* 2002;32:231-243.

3. Gault MH, Harding CE. Evaluation of i-STAT portable clinical analyzer in a hemodialysis unit. *Clin Biochem.* 1996;29:117-124.

Supplementary Table S1. Patients' demographics and baseline characteristics

Characteristic		SZC (n = 96)	Placebo (n = 99)	Total (N = 195)
Age, years	Mean	55.9	60.4	58.2
	SD	13.8	13.2	13.7
Age group, years, *n* (%)	18–50	30 (31.3)	21 (21.2)	51 (26.2)
	51–64	34 (35.4)	32 (32.3)	66 (33.8)
	65–84	32 (33.3)	45 (45.5)	77 (39.5)
	≥85	0	1 (1.0)	1 (0.5)
Sex, *n* (%)	Male	56 (58.3)	58 (58.6)	114 (58.5)
	Female	40 (41.7)	41 (41.4)	81 (41.5)
Race, *n* (%)	White	50 (52.1)	52 (52.5)	102 (52.3)
	Black or African American	10 (10.4)	8 (8.1)	18 (9.2)
	Asian	33 (34.4)	33 (33.3)	66 (33.8)
	American Indian or Alaska Native	1 (1.0)	2 (2.0)	3 (1.5)
	Other	2 (2.1)	4 (4.0)	6 (3.1)
Height, cm	Mean	166.2	165.1	165.6
	SD	9.8	9.2	9.5
Weight, kg	Mean	75.1	73.1	74.0
	SD	22.5	16.1	19.5
BMI, kg/m^2	Mean	27.0	26.7	26.9
	SD	7.1	5.4	6.3
Pre-dialysis serum K^+ concentration, mmol/L	Mean	6.0	6.0	6.0
	SD	0.4	0.4	0.4
**Dialysis history**				
Vintage, year	Mean	8.3	7.8	8.0
	SD	6.4	7.7	7.0
Access type, *n* (%)	Arteriovenous fistula	84 (87.5)	90 (90.9)	174 (89.2)

	Arteriovenous graft	7 (7.3)	3 (3.0)	10 (5.1)
	Tunneled catheter	3 (3.1)	6 (6.1)	9 (4.6)
	Right arteriovenous fistula/graft combination	1 (1.0)	0	1 (0.5)
	Missing	1 (1.0)	0 (0.0)	1 (0.5)
**Dialysis parameters**				
Dialysis adequacy, spKt/V	Mean	1.7	1.7	1.7
	SD	0.3	0.4	0.3
Urea removal rate, %	Mean	72.9	74.6	73.8
	SD	6.7	5.6	6.2
Dialysate flow, mL/min	Mean	512.0	538.5	525.5
	SD	162.8	136.0	150.0
dK$^+$ concentration, mmol/L	Mean	2.3	2.3	2.3
	SD	0.5	0.5	0.5
	Minimum, maximum	1.0, 3.0	1.0, 3.0	1.0, 3.0
Blood flow, mL/min	Mean	322.0	318.5	320.2
	SD	110.7	96.3	103.4

Safety analysis set (*N* = 195). Baseline serum K$^+$ and baseline dK$^+$ concentrations are from Visit 1 (screening).

BMI, body mass index; dK$^+$, dialysate potassium; K$^+$, potassium; SD, standard deviation; spKt/V, single-pool Kt/V; SZC, sodium zirconium cyclosilicate.

Supplementary Table S2. Proportion of patients with post-dialysis hypokalemia events post-randomization by baseline dialysate K^+ concentration in the SZC and placebo treatment groups

	SZC				Placebo			
	Baseline dK^+ concentration, mmol/L				Baseline dK^+ concentration, mmol/L			
Post-dialysis hypokalemia, n (%)	1 (*N* = 2)	2 (*N* = 67)	3 (*N* = 27)	Difference (95% CI)	1 (*N* = 1)	2 (*N* = 70)	3 (*N* = 27)	Difference (95% CI)
dK^+ = 1 vs. dK^+ = 2	2 (100.0)	60 (89.6)		0.1045 (-0.7108, 0.2520)	1 (100.0)	51 (72.9)		0.2714 (-0.6857, 0.4739)
dK^+ = 1 vs. dK^+ = 3	2 (100.0)		13 (48.1)	0.5185 (-0.3031, 0.7357)	1 (100.0)		4 (14.8)	0.8519 (-0.1282, 0.9750)
dK^+ = 2 vs. dK^+ = 3		60 (89.6)	13 (48.1)	0.4140 (0.1940, 0.6133)		51 (72.9)	4 (14.8)	0.5804 (0.3533, 0.7287)

Safety analysis set (*N* = 195). Post-dialysis hypokalemia defined as serum K^+ <3.5 mmol/L. Pairwise comparison for the proportion of any post-dialysis hypokalemia against any of two baseline dialysate K^+ categories by treatment was performed. CI for the difference was computed using the exact method, based on a score statistic.

CI, confidence interval; dK^+, dialysate potassium; K^+, potassium; SZC, sodium zirconium cyclosilicate.

Supplementary Figure S1. Pre-dialysis hypokalemia by study visit: (A) proportions of patients and (B) pre-dialysis serum K⁺ concentrations among patients with pre-dialysis hypokalemia

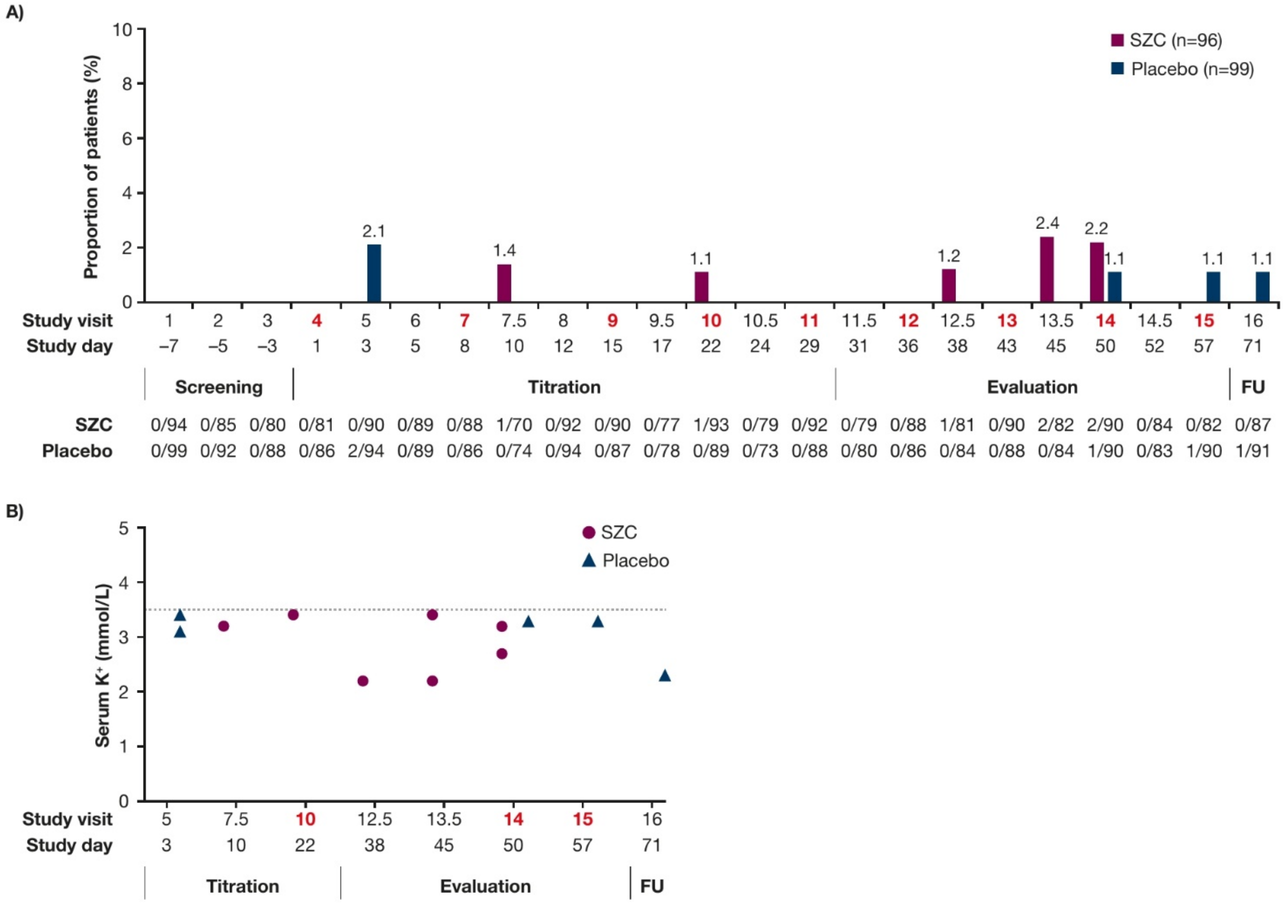

Safety analysis set (*N* = 195). Hypokalemia was defined as serum K⁺ concentration of <3.5 mmol/L (grey dashed line). Red study visits denote LIDI visits. Percentages are calculated as the number of patients with hypokalemia (serum K⁺ concentration of <3.5 mmol/L) as the numerator and the number of patients with non-missing serum K⁺ measurements as the denominator.

FU, follow-up; K⁺, potassium; LIDI, long interdialytic interval; SZC, sodium zirconium cyclosilicate.

Supplementary Figure S2. Pre-dialysis serum K^+ concentration at each study visit for patients with severe pre-dialysis hypokalemia (n = 3)

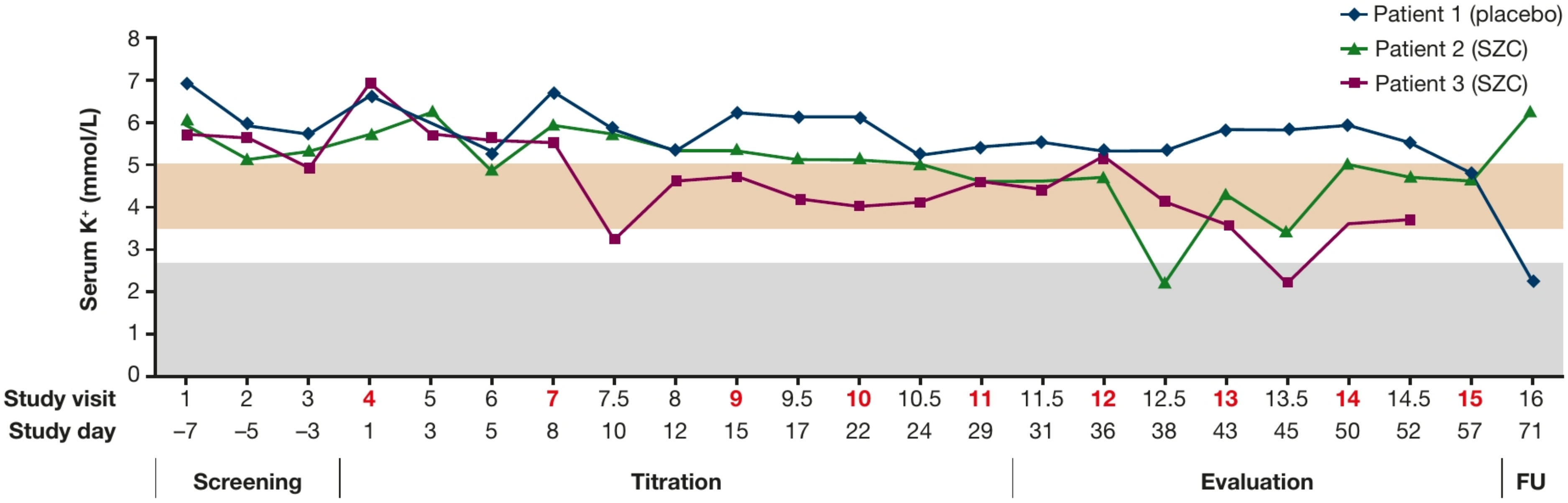

Red study visits denote LIDI visits. Severe hypokalemia is defined as serum K^+ concentration of <2.7 mmol/L (grey shaded area). Orange shaded area denotes serum K^+ concentration range between hyperkalemia (>5.0 mmol/L) and hypokalemia (<3.5 mmol/L).

FU, follow-up; K^+, potassium; LIDI, long interdialytic interval; SZC, sodium zirconium cyclosilicate.

Supplementary Figure S3. Post-dialysis hypokalemia by study visit: (A) proportions of patients and (B) median and range of post-dialysis serum K$^+$ concentration

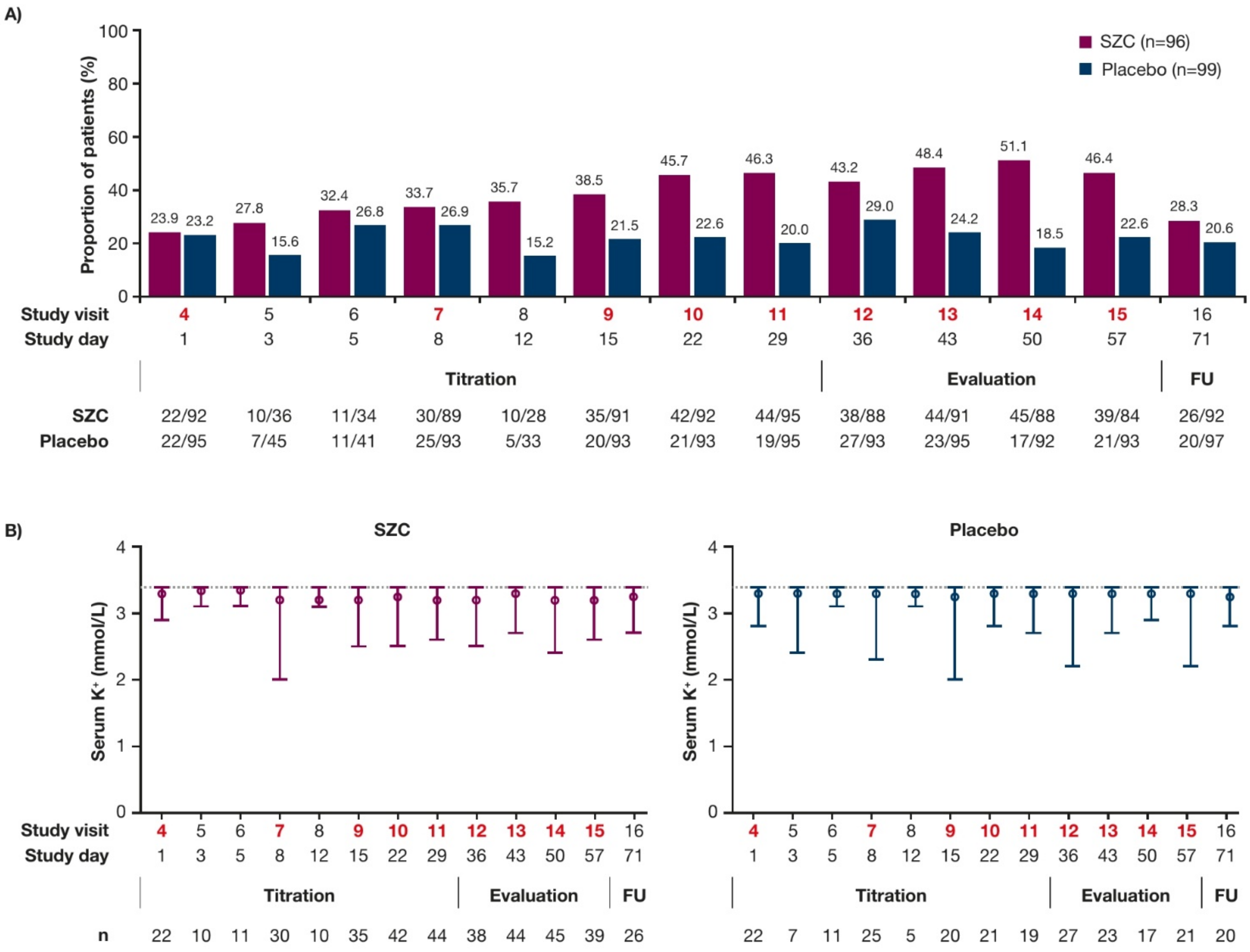

Safety analysis set (*N* = 195). Hypokalemia was defined as serum K$^+$ concentration of <3.5 mmol/L (grey dashed line). Red study visits denote LIDI visits. Percentages are calculated as the number of patients with hypokalemia (serum K$^+$ concentration of <3.5 mmol/L) as the numerator and the number of patients with non-missing serum K$^+$ measurements as the denominator. Data in B) are median values and error bars denote the minimum and maximum values.

FU, follow-up; K$^+$, potassium; LIDI, long interdialytic interval; SZC, sodium zirconium cyclosilicate.
